# Key Proteins in Rat Cerebral Cortex: Application of *Cornu aspersum* Extract as a Neuroprotective Agent in Alzheimer’s Type Dementia

**DOI:** 10.3390/molecules29225375

**Published:** 2024-11-14

**Authors:** Ventseslav Atanasov, Lyudmila Velkova, Lyubka Tancheva, Aleksandar Dolashki, Reni Kalfin, Pavlina Dolashka

**Affiliations:** 1 Institute of Organic Chemistry with Centre of Phytochemistry, Bulgarian Academy of Sciences, Acad. G. Bonchev str., bl. 9, 1113 Sofia, Bulgaria; ventseslav.atanasov@orgchm.bas.bg (V.A.); aleksandar.dolashki@orgchm.bas.bg (A.D.); pda54@abv.bg (P.D.); 2 Institute of Neurobiology, Bulgarian Academy of Sciences, Acad. G. Bonchev str., bl. 23, 1113 Sofia, Bulgaria; 3 Department of Healthcare, Faculty of Public Health, Healthcare and Sport, South-West University, Ivan Mihailov 66, 2700 Blagoevgrad, Bulgaria; 4 Centre of Competence “Clean Technologies for Sustainable Environment—Waters, Waste, Energy for a Circular Economy”, 1000 Sofia, Bulgaria

**Keywords:** Alzheimer’s disease, scopolamine, snail extract, neuroprotection, brain proteins, dementia

## Abstract

Alzheimer’s disease (AD) is the most widespread neurodegenerative disorder. Recently, it was found that mucus extract from *Cornu aspersum* has beneficial effects on memory and cognitive processes in a rat scopolamine model of AD. The present study elucidated the mechanisms of action of standardized mucus snail extract (SE) enriched with a fraction above 20 kDa on Alzheimer-type dementia in rats. Using proteomic analysis on two-dimensional polyacrylamide gel electrophoresis (2D–PAGE) on rat cortex extracts, we compared protein expression in both groups: the first group was treated intraperitoneally with scopolamine (Sco, 2 mg/kg, 11 days) and the second (Sco + SE) group was treated intraperitoneally with Sco (Sco, 2 mg/kg) and protected by SE (0.5 mL/100 g bw) applied daily orally for 11 days. Brain cortex was separated and the expressions of various proteins related to memory and cognitive functions were identified. We found that the expression of Ubiquitin carboxyl-terminal hydrolase isozyme L1, Calbindin, Vacuolar ATP synthase catalytic subunit A, Tropomyosin beta chain, 14-3-3 zeta/delta, Kinesin-1 heavy chain, and Stathmin-4 significantly differs in SE-protected rats as compared to dement animals treated only by Sco, and these brain proteins might be potential therapeutic targets for Alzheimer’s-type dementia treatment.

## 1. Introduction

Alzheimer’s disease (AD), a progressive neurodegenerative disorder, is the most common cause of dementia—a gradual deterioration of memory, thinking, behavior, and social skills. These changes affect a person’s ability to function and, at advanced stages, lead to death [1]. The causes of AD, as well as the exact mechanisms leading to neuronal death, have not yet been elucidated. It is generally accepted that selective oxidative stress in combination with a neuroinflammatory process leads to neurodegenerative changes in AD. Currently, the most important alterations identified can be explained as follows: cholinergic dysfunction, amyloid cascade, the role of the tau protein, APOE, calcium, and the involvement of the mitochondrial cascade and oxidative stress [2,3]. Other factors that affect the clinical development of AD include the vascular pathology of blood–brain barrier, which results in leakage and causes dementia [4]. Various epigenetic changes, including DNA methylation and hydroxymethylation, DNA methylation, noncoding RNA translation, and histone post-translational modifications also have been implicated in AD development [5]. AD is characterized by the loss of neurons and synapses in the cerebral cortex and some subcortical areas. This loss leads to large-scale atrophy of the affected regions, including degeneration in the temporal and parietal lobes, as well as parts of the frontal cortex [6]. It is also known that Alzheimer’s disease (AD) is a polygenic and multifactorial disease characterized by the deposition of amyloid–β (Aβ) fibrils in the brain, leading to the formation of plaques (Aβ plaques) and neurofibrillary tangles (NFTs), which are the two main pathological hallmarks of AD, and ultimately resulting in dendritic dysfunction, neuronal cell death, memory loss, behavioral changes, and organ shutdown [3].

Animal models offer valuable tools for evaluating new therapeutic strategies for the treatment of human diseases, as well as for studying the pathological mechanisms involved in these processes. In neuroscience research, rat models are preferred over mouse models because of the more complex behavioral responses in rats and their greater flexibility in dealing with novel situations [7,8].

In AD research, rats have been used for decades as important model, for instance in studies on cholinergic dysfunction and memory impairment, which played a crucial role in the development of the cholinesterase inhibitor drugs that are currently in use. In recent years, a number of transgenic rats have been reported as models for AD and new models are under development [8]. However, the use of a transgenic model has its limitations because it does not show a complete model of AD, especially sporadic forms of AD, which are prevalent. The researchers developed alternative approaches to induce AD-type dementia using various chemical drugs—colchicine, scopolamine, okadaic acid, streptozotocin, trimethyltin, aluminum trichloride, etc.—but these chemicals cause damage to certain regions of the brain–hippocampus and cortex [9]. 

Scopolamine (Sco) is used to induce cognitive dysfunction in experimental animals because it readily permeates the blood–brain barrier [10]. Sco is a muscarinic cholinergic receptor antagonist leading to memory impairment in rodents [11]. The intraperitoneal injection of Sco caused cholinergic dysfunction and impaired cognition in rats [12] and also causes an increase in amyloid-β deposition, which, along with cholinergic dysfunction, is a hallmark of AD’s molecular pathology [10]. The prolonged use of Sco is known to cause damage to the hippocampal brain cholinergic nerves and to reduce the memory of rodents in a dose-dependent manner. Sco decreased the activity of choline acetyltransferase (the enzyme responsible for acetylcholine synthesis) in the cortex of AD patients [13]. Recent studies have pointed out that Sco increases the accumulation of reactive oxygen species, inducing oxidative stress and leading to memory impairment [14]. 

At present, the search for new natural compounds for the multi-target treatment of AD is particularly relevant. In our previous research, we identified and characterized more than 40 novel peptides from *C. aspersum* mucus with potential antibacterial activity, which could provide an alternative to conventional antibiotics due to their low levels of resistance [15,16,17]. The study of a low-molecular-weight fraction of *C. aspersum* mucus using nuclear magnetic resonance (NMR) and mass spectrometry (MS) led to the identification of some secondary metabolites, such as free primary amino acids, sugars, such as glucose and sucrose, intermediates, such as fumarate, osmolites, betaine, and choline, and several other organic acids, as well as alantoin, glutathione, and some peptides with potential antioxidant and antimicrobial properties [18]. The detected choline is an important methyl group donor in various vital metabolic processes and can play a role as a precursor for the synthesis of the neurotransmitter acetylcholine, as well as participating in lipid transport and metabolism. On the other hand, some Krebs cycle intermediates, such as pyruvate, oxaloacetate and α-ketoglutarate, found in snail mucus [19], can act as energy substrates in mitochondria, but also exhibit antioxidant properties in the brain and could be effective protective molecules against oxidative stress in neuronal cells from hydrogen peroxide-mediated toxicity. Since these intermediates do not have any toxic effects, they can be used in the therapeutic treatment of chronic neurodegenerative diseases. 

The present study was designed to build on our previous research [20] related to the beneficial effects of mucus extract from *C. aspersum* snail in an experimental rat model of Alzheimer’s-type dementia. Our original results provided the first insight into the capacity of SE from *C. aspersum* to improve memory in experimental AD-type dementia. However, the complex mechanisms of this disease deserve more in-depth studies.

Hence, the goal of present study was to shed additional light on the capacity of SE to change the expression of some important brain proteins that could be potential therapeutic targets for the treatment of AD, using a proteomic analysis of two-dimensional gel electrophoresis (2D-PAGE), MALDI-MS and MALDI-MS/MS, and bioinformatics. 

## 2. Results

### 2.1. Preparation of Mucus Extract

The crude mucus was collected from snails grown in Bulgarian eco farms using the patented technology presented in [21]. The resulting crude mucus extract was homogenized and centrifuged to remove coarse impurities. After several filtration steps (also using the patented technology), the native mucus extract was obtained as described in [15,16,17]. The protein fraction with components with MW above 20 kDa, obtained using a concentration of native mucus extract on polyethersulfone membrane with a pore size 20 kDa (Microdyn Nadir^TM^ from SterliTech Corporation, Goleta, CA USA), was added to the purified initial mucus; the ratio of the two fractions was 1:1, as described in our previous study [20]. In this way, a combined extract of snail mucus was obtained, which was designated as snail extract (SE) with a protein concentration of 1.15 mg/mL. This was used for the treatment of the laboratory animals, as described in [20]. In our previous study, the beneficial effects of this SE on the Alzheimer’s-type dementia experimental model in rats were established; the SE possessed moderate antioxidant properties, demonstrated acetylcholinesterase-inhibitory activity, and was shown to modulate monoamine content in memory-related brain structures [20].

### 2.2. Treatment of Laboratory Animals Using Mucus Extract and Scopolamine

To induce AD-type dementia, laboratory rats were injected intraperitoneally with Scopolamine hydrobromide, designated as Sco. Male adult Wistar rats were used as laboratory animals. They were divided into two groups: the first (Sco group) was treated for 11 days intraperitoneally (Sco, 2 mg/kg) and the second (Sco + SE) was treated intraperitoneally with Sco (Sco, 2 mg/kg) and SE (0.5 mL/100 g bw), applied orally. 

At the end of the experiment, the laboratory animals were euthanized and decapitated, and the prefrontal cortex was isolated from their brains. After homogenization and centrifugation (at 10,000 rpm for 30 min at 4 °C) of the cortex samples, the protein concentrations of the supernatants were evaluated using the Bradford method and the probes were prepared for two-dimensional gel electrophoresis (2D-PAGE).

### 2.3. Identification of Proteins on 2D-Gel Electrophoresis Using Mass Spectrometry and Bioinformatics

**Figure 1:**
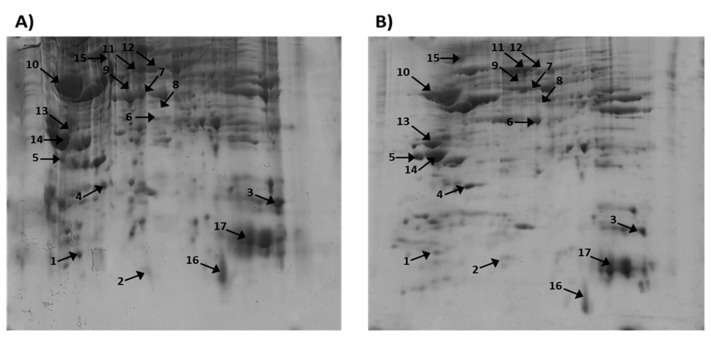
Two-dimensional-PAGE of cellular proteins from the rat cortex: (**A**) treated with Sco and (**B**) treated with Sco + SE (pI 3–10, 12.0% SDS-polyacrylamide gels–Coomassie Brilliant Blue G-250 staining). Note: Annotated numbers refer to the identification of the proteins in Table 1.

**Figure 2:**
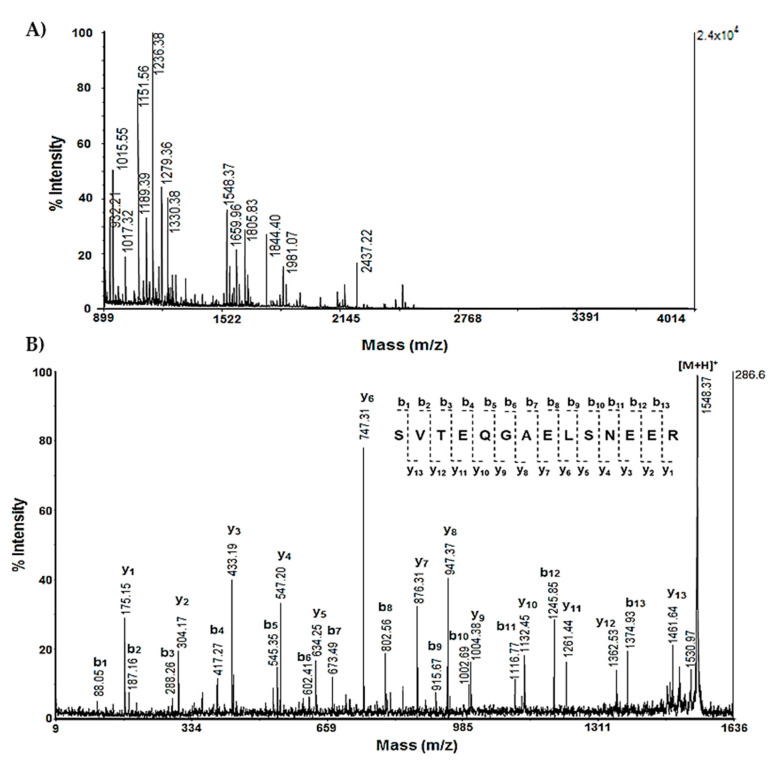
Mass spectrometric analysis of protein on spot A14 from 2D-PAGE: (**A**) MS spectrum of peptides obtained after the trypsin digestion of spot 14 with experimental MW 27.754 kDa and pI 4.73; (**B**) MS/MS spectrum from MALDI-TOF-TOF analysis of peptide at *m*/*z* 1548.37 from spot 14: 1433Z_RAT.

**Figure 3:**
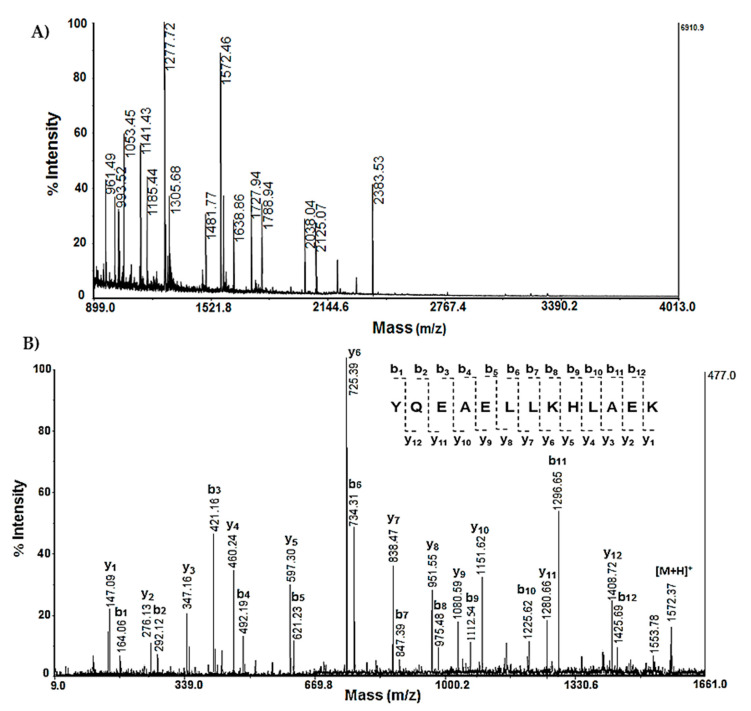
Mass spectrometric analysis of protein on spot A 16 from 2D-PAGE: (**A**) MS spectrum of peptides obtained after trypsin digestion of spot 16 with experimental MW 22.073 kDa and pI 5.76; (**B**) MS/MS spectrum from MALDI-TOF-TOF analysis of peptide at *m*/*z* 1572.46 from spot 16: STMN4_RAT.

Using two-dimensional gel electrophoresis (2D-PAGE) coupled with the MelanieTM Coverage 9.2 software, as well as the MALDI-MS and MALDI-MS/MS experiments, we analyzed the rat cortex extracts, which were divided into two groups: the first group was treated intraperitoneally with scopolamine (Sco, 2 mg/kg, 11 days) and the second (Sco + SE) group was treated intraperitoneally with Sco (Sco, 2 mg/kg) and protected by SE (0.5 mL/100 g bw), administered orally every day for 11 days. The proteins in the rat cerebral cortex were separated via 2D-electrophoresis and stained with Coomassie brilliant blue. A comparison of the 2D-PAGE images obtained using MelanieTM Coverage 9.2 software showed that a number of proteins changed their expression (Figure 1A,B). After the high-resolution scanning of the gels, the differences in protein expression between the Sco and Sco + SE group, as well as the molecular weight (MW) and isoelectric point (pI) of the corresponding proteins, were determined through computer-aided 2D image analysis, carried out using Melanie^TM^ Coverage 9.2. The software calculated the pixel intensities of all spots. A prediction of the proteins obtained from the 2D-PAGE spots of the cortex was made using the obtained data regarding their molecular weight (MW) and isoelectric point (pI) and a comparison with the database UniProt, taxonomy *Rattus norvegicus*, brain (https://www.uniprot.org/, accessed on 12 September 2024). In this way, we found proteins that perform different functions, such as structural, antioxidant, metabolic, and signaling, as well as those involved in the folding of other proteins, etc. The key proteins, along with their change in expression in Sco and Sco + SE rats, are presented in Table 1. Selected spots from the polyacrylamide gels were excised, subjected to trypsin digestion, and prepared for subsequent mass spectrometric analysis. After the extraction of peptides from each protein spot, they were subjected to MALDI-MS analysis (Figure 2A and Figure 3A).

The protein identification was performed using MASCOT software (http://www.matrixscience.com, accessed on 12 September 2024), based on the MALDI-MS analysis of peptides extracted from the gel after the triptic digestion of protein spots. The monoisotopic *m*/*z* values of the peptide mass fingerprint were compared to known proteins in the Swiss-Prot database using the following search parameters: Peptide Mass Fingerprint; Database: SwissProt; Taxonomy: Rattus; Enzyme: Trypsin; Allow up to: 1–3 missed cleavages; Monoisotopic; Mass Values MH^+^; Peptide tolerance: ±1.2 Da and a probabilistic score at *p* < 0.05. The theoretical and observed pI and MW values were in good agreement. Proteins detected in Mascot were checked against the UniProtKB online database (http://www.uniprot.org, accessed on 12 September 2024) in order to verify their localization and characterize their functions. For example, the MS spectrum of peptides obtained after the tryptic digestion of proteins obtained as protonated molecular ions [M+H]^+^ from spot A14 of 2D-PAGE is shown in Figure 2A. The analysis of the MS spectrum using MASCOT fingerprint showed 42% protein sequence coverage, corresponding to 14-3-3 protein zeta/delta (1433Z_RAT) and an expected E value = 2.1 × 10^−12^. After the interpretation of fragment ions in the MS/MS spectrum of the peptide [M+H]^+^ at *m*/*z* 1548.37, the obtained sequence (SVTEQGAELSNEER) also confirmed the presence of 14-3-3 protein zeta/delta (1433Z_RAT).

Another protein that significantly changes its expression is Stathmin-4 (STMN4_RAT), which was determined with MW 22 073 Da at pI 5.7 and found to be present at site A16 via mass spectrometric analysis (Figure 3A,B). This was confirmed by MS analysis following the use of Mascot Peptide Fingerprint and interpretation of the MS/MS spectrum.

Peptide sequences generated from the MS/MS spectra were added to BLAST (https://blast.ncbi.nlm.nih.gov/Blast.cgi, accessed on 15 September 2024) to confirm the corresponding proteins. Using this method, the eight proteins shown in Table 2 were successfully confirmed. The amino acid sequences of tryptic peptides of several protein spots from 2D-PAGE, determined by MS/MS analyses, are presented in Table 2.

## 3. Discussion

Because of the multifactorial nature of AD, it is difficult to reveal the exact mechanisms underlying this disorder. It is likely that oxidative stress triggers the disease by increasing the production and aggregation of Aβ amyloid peptides, facilitating τ-hyperphosphorylation and its aggregation into NFTs [22]. The abnormal accumulation of β-amyloid promotes ROS formation through the activation of NMDA receptors, which is also of importance [23]. Studies have shown that amyloid β interacts with cholinergic receptors, affecting their function [24]. The scopolamine model is a widely used and well-established experimental model for inducing Alzheimer’s-like symptoms, especially in studies of cognitive impairment, as it effectively mimics key aspects of Alzheimer’s disease, such as memory deficits, making it a valuable tool in preclinical research [25,26,27]. Scopolamine is known to increase AChE activity, decrease ACh levels, and inhibit choline acetyltransferase activity, thereby inducing neuroinflammation through high levels of oxidative stress and the impairment of antioxidant defense mechanisms, in addition to increasing tau protein levels and amyloid deposits. Its administration in animal models has been shown to induce marked histopathological changes in the cerebral cortex, including neuronal degeneration [28]. The damaging mechanisms of action of scopolamine are summarized in the scheme published by Kaur et al. (2015) [28]. 

Recently, our research showed that mucus extract from *C. aspersum* demonstrated complex therapeutic potential under conditions of scopolamine-altered behavior, biochemistry, and gene expression in rat brain models of Alzheimer’s-type dementia. The obtained results showed that the snail extract possessed moderate antioxidant properties, demonstrated acetylcholinesterase-inhibitory activity, and modulated monoamine content in memory-related brain structures. The confirmation of these results via principal components analysis demonstrated a very close similarity between the Sco + snail group and control groups, supporting the restorative effect of SE in demented animals. Moreover, we reported also a very pronounced effect of the snail extract on gene expression [20].

The results obtained through a proteomic analysis of the 2D-PAGE of the cortex extract of rats treated with scopolamine and rats treated with Sco + SE showed a change in the expression of a number of proteins with diverse functions—structural, regulatory, metabolic, signaling, antioxidant, etc.,—involved in various aspects of AD progression. The functions and the biological role of key proteins identified from the rat cortex are summarized in Table 3 based on their protein function in rat brain, as been previously published in many studies.

Cytoskeletal proteins are important for the construction and functioning of the cytoskeleton. They include actin, tubulin, and GFAP (Glial Fibrillary Acidic Protein). A number of studies have shown that the actin cytoskeleton plays a key role in synaptic function and plasticity, since it takes part in different aspects of the coordinated machinery that modulates synaptic transmission [35]. 

Our results showed an increase in the expression of β-actin in the Sco-treated animals and a decrease in the rats treated with scopolamine and SE together. The increased levels of β-actin in the demented rats likely reflect an increase in the production of the plaque-forming Aβ peptide that is deposited outside neurons. Thus, actin may be positioned at the crossroads between the amyloid cascade and synaptic dysfunction and is likely a key link between them in the pathogenesis of AD. 

Tubulin is the major constituent of microtubules, which are composed of α–β-tubulin heterodimers forming linear protofilaments that form a hollow polar cylinder. Microtubules (MT) are essential components of the cytoskeleton of the cell, which has locomotory functions. In the cell, MTs are among the most complex structures in terms of both their chemical and functional properties [38]. Our studies showed a decrease in the expression of tubulin in rats with Alzheimer’s-type dementia and an increase in the subsequent SE-based treatment of the animals with dementia. 

GFAP is a key protein responsible for the cytoskeletal structure of glial cells and for maintaining their mechanical strength, regulating astrocyte morphology and function, as well as maintaining neighboring neurons and the blood–brain barrier. This protein is not found outside the CNS [37]. Our studies showed no significant differences in GFAP expression in the two groups of rats. This may be due to the Alzheimer’s-type dementia being at an earlier stage in the Sco group.

Proteins involved in signal pathways mediate significant events in the cellular life cycle, and they are closely associated with AD development. Cytoskeletal protein regulators, which are often part of signaling cascades, also play a key role in AD progression. Such proteins include 14-3-3, stathmin, kinesin, cofilin, and tropomyosin. The protein 14-3-3 is an adapter protein involved in the regulation of a wide range of general and specialized signaling pathways [43]. 

Our studies showed a twofold increase in the expression of 14-3-3 in the Sco group of rats compared to the group treated with Sco and SE together. These results correlate with the literature data suggesting an important role of 14-3-3 in regulating MT dynamics by phosphorylating the MT-associated protein tau. Moreover, the 14-3-3 protein is likely to be one of the key components of the mechanism of action by which snail extract improves memory in Sco-affected demented animals. 

The protein Stathmin-4 (STM-4) is a member of the Stathmin family of phosphoproteins expressed in the nervous system, which binds to tubulin and destabilizes MTs. It is known that stathmins play a crucial role in neuronal differentiation and plasticity, and their dysregulation is associated with various brain and neurodevelopmental disorders. The function and biological role of STM-4 are specified in Table 3. In this context, our results showed a multi-fold increase in the expression of stathmin in Sco-treated animals and very low levels in the group of rats treated with Sco and SE together. This suggests a positive effect of SE on the cytoskeleton of neurons in general. In our studies, a moderate increase in the expression levels of kinesin-1 heavy chain was observed in the Sco group of rats, which confirms the literature data [45,48]. 

Cofilin is a ubiquitously expressed protein. Results obtained from multiple studies suggest its critical role in the pathogenesis of AD [31]. Our studies showed a slightly increased expression of cofilin in the animals treated with Sco compared to those co-treated with Sco and SE. 

Tropomyosin (Tm) was shown to be an integral component of the neurofibrillary pathology (NFP) of AD. Studies have shown that a protein other than tau, sneurofilaments, MAP2, or ubiquitin, which is antigenically related to tropomyosin, is involved in the abnormal filaments characteristic of the NFP of AD. However, the exact role of Tm in AD pathology is still not well understood [41]. 

Our studies showed a fold reduction in Tm expression levels in rats co-treated with Sco and SE compared to the scopolamine group. This suggests the key role of Tm in AD. The mechanisms of this action remain to be elucidated. 

Obligate metabolic enzymes participate in biochemical pathways and cycles in all eukaryotic cells. They could be used to determine the metabolic state of brain cells or brain tissue in different pathologic and physiologic conditions. An important representative of these enzymes is malate dehydrogenase (MDH). The functional significance of changes in MDH levels in AD is not yet known [34], but it is known that the enzyme plays an essential role in the malate–aspartate shuttle and the citric acid cycle, which are important for the mitochondrial supply of NADH for oxidative phosphorylation. Our data showing a decrease in MDH expression in the Sco group of rats suggest disturbances in the neuronal metabolism in AD-type dementia. Snail mucus has a positive effect on the metabolism.

Heat-shock proteins have many functions as molecular chaperons in cells and work as an integrated network, participating in the folding of newly synthesized polypeptides, refolding metastable proteins, protein complex assembly, protein aggregate dissociation, and the degradation of misfolded proteins. In addition, they also play important roles in cell signaling transduction, the cell cycle, and apoptosis regulation [49]. Heat-shock protein 60 (Hsp60) is a chaperone that is localized in the mitochondria and is involved in the correct folding of proteins. Many studies have shown that Hsp60 is localized outside of mitochondria, such as in the cytosol, in extracellular vesicles, or on the cell surface. The role of Hsp60 in AD is still unclear. Furthermore, the extracellular release of Hsp60 increases the production of other pro-inflammatory factors and promotes neuronal cell death. The hyperactivation of microglia in response to certain negative factors contributes to the progression of several neurodegenerative diseases, including AD [39]. 

Our results showed an increase in the expression of Hsp60 in the animals treated with Sco and SE together, which is evidence of its protective role. 

Ubiquitin carboxyl–terminal hydrolase isozyme L1 (UCH L1) protein belongs to the Ubiquitin system, which is a non-lysosomed protein degradation pathway [50]. It is an important cell regulation system and may be regulated at THE protein degradation level but not at the transcriptional level [43]. 

Our research showed a decrease in the expression of UCH L1 in the Sco group of rats and an increase in the group of animals treated with Sco and SE together. Restoring Uch-L1 activity may represent a new therapeutic strategy for AD.

The proteins involved in Ca^2+^ homeostasis and cell bioenergetics that we determined are calbindin, creatine kinase, and ATP synthase. The regulation of intracellular calcium homeostasis is a very complex mechanism that is vital to several cellular pathways and is thus involved in cell survival and death. Cellular bioenergetics forms the basis of the life processes in all cells under physiological and pathological conditions. Calbindin (CB) buffers cytosolic calcium. It can stimulate membrane Ca^2+^-ATPase and 3’,5’-cyclic nucleotide phosphodiesterase. CB has a critical role in preventing neuronal death as well as maintaining calcium homeostasis. Although a significant reduction in CB expression was observed in the brains of AD mice and humans, it is not known whether these changes contribute to AD-related dysfunction. Experiments have shown that the removal of CB from the amyloid precursor protein presenilin in transgenic mice worsens the pathogenesis of AD, suggesting a critical role of CB in the pathogenesis of AD [33]. Our results confirm the literature data, showing a decrease in CB expression in animals with dementia compared to the rats treated with Sco and SE together. Mitochondrial bioenergetics is not only essential in ATP synthesis but is also important in the regulation of several other mitochondrial and cellular processes that are affected in AD. The dysregulation of these processes can further increase the extent of mitochondrial dysfunction and cellular damage induced by dysfunctional bioenergetics, creating a vicious cycle that will ultimately lead the cell to apoptosis [40]. Our studies showed a two-fold reduction in the expression of ATP synthase in the rats treated with Sco compared to the group of animals treated with Sco and snail extract together. These results direct attention to the potential of this enzyme as a therapeutic target for pharmacological preparations aimed at preventing mitochondrial dysfunction in AD and, accordingly, inhibiting apoptosis in neurons.

Because some brain hemoglobin may be derived from the peripheral circulation due to a compromised blood–brain barrier often seen in the brains of elderly individuals and those with AD, research by Chuang and colleagues suggests that the genesis of some plaques may be a consequence of the prolonged accumulation of amyloid at sites of vascular injury [51]. Our results showed a fold-increase in the expression of Hemoglobin (Hb) in the Sco group of rats compared to the insignificant expression observed in the group of animals treated with Sco and SE together. This is probably indirect evidence of the close relationship between Hb and Aβ in the progression of AD and the improvement in the disease after treatment with snail mucus. On the other hand, the increased expression of the Hb α subunit is controversial and is likely to reflect the impaired blood–brain barrier.

Antioxidant proteins also play an important role in nervous system diseases and physiological conditions. These include thioredoxin and Cu-Zn Superoxide dismutase (CuZn-SOD). 

Thioredoxin (Trx) plays a role in the reversible S-nitrosylation of cysteine residues in target proteins and thereby contributes to the intracellular nitric oxide response. In the brain, Trx is activated upon ischemia/reperfusion and appears to play an important role not only in oxidative stress but also in signal transduction. In vitro and in vivo experiments have demonstrated that the enhancement of endogenous Trx expression and administration of exogenous Trx inducers play neuroprotective roles in AD, such as inhibiting oxidative stress, inflammation, and apoptosis, and activating survival signaling pathways. These findings suggest that increasing Trx expression and activity may serve as a strategy to delay AD progression [29]. 

The results of the proteomic analysis show large differences in the expression of cortex proteins in the two groups of rats with Alzheimer’s-type dementia: the first group treated intraperitoneally with scopolamine (Sco, 2 mg/kg, 11 days) and the second (Sco + SE) group treated intraperitoneally with Sco (Sco, 2 mg/kg) and protected by SE (0.5 mL/100 g bw), applied daily orally for 11 days. The presented results show the dysregulation of Ubiquitin carboxyl-terminal hydrolase isozyme L1, Calbindin, Vacuolar ATP synthase catalytic subunit A, Tropo-myosin beta chain, 14-3-3 zeta/delta, Kinesin-1 heavy chain, and Stathmin-4, which could be potential drug targets. This study builds on the results obtained in our previous study [20] and elucidates some aspects of the mechanism of action of SE in protecting against Alzheimer’s-type dementia. In [20], we found that SE demonstrated acetylcholinesterase-inhibitory activity, moderate antioxidant properties, and the ability to modulate the monoamines content in brain structures related to memory. We hypothesize that the snail extract probably activates protein isoenzymes that are involved in neuroprotective mechanisms under conditions of oxidative stress.

## 4. Materials and Methods

### 4.1. Snail Extract

The mucus was collected from *C. aspersum* snails grown in Bulgarian eco-farms according to a patented technology, without causing suffering to the snails; this process is described in [21]. The resulting crude mucus extract was purified through several steps of centrifugation and filtration, as previously reported [15,16,17]. The purified native mucilage extract was used for the obtained fractions containing bioactive molecules with an MW above 20 kDa via pressure ultrafiltration with 20 kDa pore size polyethersulfone membrane filters (Microdyn Nadir™ from STERLITECH Corporation, Goleta, CA, USA). After a mixture of 50% purified initial mucus and a 50% fraction of MW above 20 kDa was created, snail mucus extract (SE) with a concentration of 1.15 mg/mL (according to the Bradford method, [52]) was obtained. The use of this non-invasive technique ensures that SE containing intact bioactive compounds is obtained. The SE was used for the treatment of the laboratory animals, as described in [20].

### 4.2. Laboratory Animals

Male adult Wistar rats ranging in age from six to eight weeks old were purchased from a local vendor (Erboj, Slivnica, Sofia, Bulgaria). Rats were housed three per cage under constant laboratory conditions (25 ± 3 °C; 12/12 h light/dark cycle; food and water available ad libitum). A five-day habituation period took place before starting the experiment. 

All experiments were performed in strict accordance with the national regulations and European Communities Council Directive (86/609/EEC) and the “Principles of laboratory animal care” (NIH publication No. 85–23) concerning the protection of animals used for scientific and experimental purposes. The research on laboratory animals was approved by the Committee of Bioethics at the Institute of Neurobiology, Bulgarian academy of Sciences.

### 4.3. Scopolamine Induced Dementia

The rats were injected with Scopolamine hydrobromide (2 mg/kg, intraperitoneally- i.p.) for 11 consecutive days. This was carried out in order to induce AD-type dementia. The dose of scopolamine was determined in our previous studies [20]. 

### 4.4. Treatment of the Laboratory Animals

The animals were divided into two groups (six rats in each group) as follows: (1) Sco-treated control group and (2) Sco + SE-treated group. Sco and Sco + SE groups were injected with scopolamine hydrobromide at a dose of 2 mg/kg. The Sco + SE group received snail extract from *C. aspersum* orally (0.5 mL/100 g bw), and the Sco group received dH_2_O orally (0.5 mL/100 g bw) instead SE. Sco was dissolved in distilled water ex tempore before each administration. SE was applied 1h before the Sco injection daily for 16 days (5 days before and 11 days simultaneously with Sco), as described previously [20].

### 4.5. Extraction of Proteins from the Rat Brain

Rat brains were rapidly removed after the animals were euthanized via mild CO_2_ inhalation followed by decapitation. The samples from rat cortex (about 3–4 g) were washed with 10 mL of ice-cold PBS buffer (0.2 g KCl, 8 g NaCl, 1.44 Na_2_HPO_4_, 0.24 g KH_2_PO_4_). Then, the samples were homogenized in 0.1 M phosphate buffer, pH 7.8, containing protease inhibitors (Calbiochem Protease Inhibitor Cocktail Set 111). After complete homogenization, the samples were centrifuged at 10,000 rpm for 30 min at 4 °C. Of the pure supernatants obtained, protein concentrations were assessed using a Bradford Protein Assay Kit (Bio-Rad, Hercules, CA, USA). The supernatants were used for examination with 2D–PAGE.

### 4.6. Two-Dimensional-PAGE Analysis

A 2D-PAGE analysis was conducted to examine changes in the expression of the proteins, as described in [53]. After the protein concentration of the supernatants was determined with the Protein Assay Kit (Bio-Rad Laboratories, Inc., Hercules, CA, USA), approximately 160 μg of the protein was mixed with an IPG rehydration buffer, containing 8 mol/L urea, 2% *w*/*v* CHAPS, and 0.3% dithiothreitol (DTT), at a final volume of 125 μL. The strips were rehydrated for 12 h and focused (IEF) using the PROTEAN^®^ i12™ IEF System (Bio-Rad Laboratories, Inc.). At the end of the IEF program, the IPG strips were equilibrated in a 50 mmol/L Tris-HCl solution, pH 8.8, containing 6 mol/L urea, 30% glycerol, 2% SDS, and 1% DTT, for 10 min; after this, the solution was replaced with the same solution, but the DTT was exchanged with 5% iodoacetamide. The strips were placed on home-casted vertical 12.0% SDS-PAGE gels. Staining was performed using Coomassie Brilliant Blue G-250 Dye (CBB G-250, Thermo Fisher Scientific, Waltham, MA, USA).

### 4.7. Trypsin Digestion of Protein Spots

The trypsin digestion of proteins from 2D PAGE was conducted as described by Rosenfeld et al. [54]. After two washes with 150 μL of 200 mmol/L ammonium bicarbonate in 50% ACN/MQ (30 min at 30 °C), discolored gel pieces were dried in a SpeedVac (Thermo Savant, Holbrook, NY, USA). A total of 10 μL digestion buffer was added to the dried gel spots. It contained 50 mM ammonium bicarbonate, pH 7.8, and modified trypsin per microliter (Promega). The tubes were kept on ice for 45 min to allow the gel pieces to be completely soaked with the protease solution. The digestion reaction was performed overnight at 37 °C. Then, the supernatants were recovered and the resulting peptides were extracted twice with 35 μL of 60% ACN/0.1% DIEA. The extracts were pooled and dried in the speed vac machine (Labconco^®^ Centrivap Concentrator, Kansas City, MO, USA). The extracted peptides were re-dissolved in 10 μL of a 0.1% formic acid and matrix solution (a saturated solution of alpha cyano-4-hydroxycinnamic acid in acetonitrile/water 50:50 with 0.1% TFA for peptide measurements of <10 kDa) and spotted on the MALDI plate.

### 4.8. Mass Spectrometric Analyses (MS and MS/MS)

After tryptic digestion, the proteins were analyzed using an Autoflex™III, high-performance MALDI-TOF and TOF/TOF system (Bruker Daltonics, Billerica, MA, USA). This is a highly effective system using a 200 Hz frequency-tripled Nd–YAG laser, whose wavelengths operates at 355 nm. Samples were prepared by mixing 2.0 μL of the sample with a 2.0 μL matrix solution (7 mg/mL α-cyano-4-hydroxycinnamic acid (CHCA) in 50% acetonitrile (ACN) containing 0.1% trifluoroacetic acid (TFA); then, the sample was placed on a stainless plate with 192 pits. An MS mode and collision energy of 4200 were used after a total of 3500 shots and after drying the samples at room temperature. The mass scale was calibrated using a solution of protein standards. The external calibration of the apparatus was performed with a mixture of angiotensin I, glu-fibrinopeptide B, ACTH (1–17), and ACTH. 

### 4.9. Identification of Proteins

The identification of proteins from MALDI/MS spectra was achieved using different protein databases, obtained using a Peptide Mass Fingerprints search with MASCOT http://www.matrixscience.com, accessed on 15 September 2024 (MASCOT server, Matrixscience, London, UK). The parameters were as follows: Type of search: Peptide Mass Fingerprint; Database: SwissProt; Taxonomy: Rattus; Enzyme: Trypsin; Allow up to: 1–3 missed cleavages; Monoisotopic; Mass Values MH^+^; Peptide tolerance: 1.2–2.5 and a probabilistic score at *p* < 0.05. the proteins detected in Mascot were checked against the UniProtKB online database (http://www.uniprot.org, accessed on 15 September 2024). The amino acid sequences revealed through manual interpretation of the MS/MS spectra were in alignment with those in the Swiss Prot and NCBI BLAST databases (https://blast.ncbi.nlm.nih.gov/Blast.cgi, accessed on 15 September 2024). An evaluation of gel maps was conducted by Melanie^TM^ Coverage 9.2. Differences in protein expression in rat cortex between Sco control and Sco + SE-treated animals, as well as the molecular weight (Mw) and isoelectric point (pI) of corresponding proteins, were determined through computer-aided 2D image analysis, carried out using Melanie^TM^ Coverage 9.2 software.

## 5. Conclusions

The present study, for the first time, shows significant differences in the protein ex-pression of Ubiquitin carboxyl-terminal hydrolase isozyme L1, Calbindin, Vacuolar ATP synthase catalytic subunit A, Tropomyosin beta chain, 14-3-3 zeta/delta, Kinesin-1 heavy chain, and Stathmin-4, detected via proteomic analysis in two groups of rats—the first group treated only with Sco, and the second group treated with Sco but protected with SE. Some of these proteins may be potential drug targets in the treatment of Alzheimer’s dementia. Our future work will expand and build on these studies to provide a more comprehensive view of the protective mechanisms of SE in the cortex and hippocampus of animal models of Alzheimer’s disease at the molecular level.

## Figures and Tables

**Table 1:** Identified key cortex proteins in two groups of rats, characterized by their MW, pI, and expression, calculated using Melanie Coverage 9.2 software and confirmed using MASCOT software (MASCOT server, Matrixscience, London, UK, available at https://www.matrixscience.com, accessed on 12 September 2024) based on the performed MALDI-MS analyses. The expression of proteins extracted from the cortex of the first group of treated rats (Sco-treated) corresponds to the blue bar (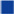) and the expression of proteins extracted from the second group (Sco + SE-treated animals) is represented by the green bar (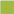).

No. Spot Cortex	Protein Name (UniProtKB)	Volume (Pixels) Sco	Volume (Pixels) Sco + SE	MW Exp.	pI Exp.	MW Theor	pI Theor	Expression
1	Thioredoxin; P11232 (THIO_RAT)	6,050,118	5,441,182	15.081	4.48	11.673	4.80	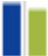
2	Superoxide dismutase [Cu-Zn]; P07632 (SODC_RAT)	6,261,736	7,118,268	18.158	5.51	15.912	5.88	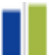
3	Cofilin-1; P45592, (COF1_RAT)	1,157,164	1,101,119	20	7.46	18.533	8.22	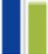
4	Ubiquitin carboxyl-terminal hydrolase isozyme L1; Q00981 (UCHL1_RAT)	2,204,242	3,605,218	22	4.9	24.838	5.14	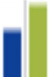
5	Calbindin; P07171 (CALB1_RAT)	8,396,846	11,756,730	28	3.94	29.994	4.71	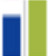
6	Malate dehydrogenase, cytoplasmic; O88989 (MDHC_RAT)	12,166,374	15,772,607	36	5.63	36.483	6.16	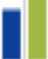
7	β –Actin; P60711 (ACTB_RAT)	18,729,148	13,744,467	48	5.35	41.737	5.2	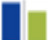
8	Creatine Kinase B-type; P07335 (KCRB_RAT)	375,048	448,176	40	5.52	42.725	5.4	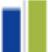
9	Glial Fibrillary Acidic Protein; P47819 (GFAP_RAT)	582,304	583,050	53	5.17	49.957	5.02	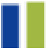
10	Tubulin α-1 chain; Q6P9V9 (TBA1B_RAT)	22,419,986	7,427,465	53	4.35	50.152	4.94	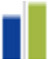
11	60 kDa heat shock protein, mitochondrial; P63039 (CH60_RAT)	10,059,479	13,101,991	71	5.44	60.955	5.91	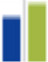
12	Vacuolar ATP synthase catalytic subunit A; P50516 (VATA_RAT)	2,211,577	3,933,414	73	5.28	68.326	5.41	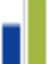
13	Tropomyosin beta chain P58775 (TPM2_RAT)	2,502,851	451,024	32.817	4.66	41.253	4.45	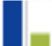
14	14-3-3 protein zeta/delta P63102 (1433Z_RAT)	15,894,376	7,086,121	27.754	4.73	33.326	4.46	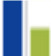
15	Kinesin-1 heavy chain Q2PQA9 (KINH_RAT)	4,906,008	3,993,752	109.463	6.06	126.318	5.51	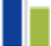
16	Stathmin-4; P63043 (STMN4_RAT)	39,377,068	10,224,790	22.073	5.76	17.650	6.42	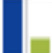
17	Hemoglobin subunit alpha-1/2; P01946 (HBA_RAT)	27,582,716	2,551,968	15.319	7.82	21.167	7.7	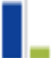

**Table 2:** Amino acid sequences of tryptic peptides of several spots from 2D-PAGE images, determined by MS/MS analyses.

Spot No.	AAS of Peptide	Mass [M+H]^+^	Spot No	AAS of Peptide	Mass [M+H]^+^
6	DLDVAVLVGSMPR	1371.71	14	MKGDYYR	932.21
	FVEGLLPNDFSR	1393.68		DSTLIMQLLR	1189.39
	SQIALKLGVTADDVK	1558.84		KEMQPTHPIR	1236.38
	VIVVGNPANTNCLTASK	1700.89		SVTEQGAELSNEER	1548.37
	GEFITTVQQRGAAVIK	1719.94		LAEQAERYDDMAACMK	1844.40
7	EITALAPSTMK	1161.61	15	KMEENEK	908.29
	IWHHTFYNELR	1515.74		YQQEVDRIK	1177.35
	SYELDPGQVITIGNER	1790.87		EYELLSDELNQK	1479.48
	YPIEHGIVTNWDDMEK	1946.89		TQMLDQEELLASTRR	1791.35
	VAPEEHPVLLTEAPLNPK	1954.06		GLEETVAKELQTLHNLR	1949.51
10	YMACCLLYR	1135.50	16	MTLAAYKEK	1053.45
	QLFHPEQLITGK	1410.77		EAHLAAMLER	1141.43
	SIQFVDWCPTGFK	1527.73		RKYQEAELLK	1277.72
	IHFPLATYAPVISAEK	1756.67		YQEAELLKHLAEK	1572.46
	EDAANNYARGFYTIGK	1790.87		MKELPLVSLFCSCFLSDPLNK	2383.53
	KKMQMLK	907.24		MFAAFPTTK	1013.31
	HIAEDSDR	941.24		LRVDPVNFK	1087.42
13	AEFAERSVAK	1107.91	17	IGGHGGEYGEEALQR	1572.47
	LEEAEKAADESER	1475.70		TYFSHIDVSPGSAQVK	1735.55
	LEEAEKAADESERGMK	1791.71		AADHVEDLPGALSTLSDLHAHK	2296.75
	TIDDLEDEVYAQKMKYK	2088.89			

**Table 3:** Functions and biological role of key proteins determined via proteomic analysis of two groups of rats: the first group was treated intraperitoneally with Sco (2 mg/kg, 11 days) and the second group was treated intraperitoneally with Sco (Sco, 2 mg/kg) and protected by SE (0.5 mL/100 g body weight) administered orally daily for 11 days.

Spot No.	Protein Name	Molecular Function	Biological Process
1	Thioredoxin P11232 (THIO_RAT)	Thioredoxin (Trx) inhibits caspase-3 activity by nitrosylating its active Cys site.	Trx has multiple biological functions, including protective cellular mechanisms against oxidative stress and cytokine-induced damage. The elevation of endogenous Trx expression and transfer of exogenous Trx-inducers activate pro-survival signaling pathways, thus playing a neuroprotective role in AD [29].
2	Superoxide dismutase [Cu-Zn] P07632, SODC_RAT)	CuZnSOD catalyzes the dismutation of the superoxide anion radical to O_2_ and H_2_O_2_.	It is suggested that CuZnSOD plays a key role in the antioxidant protection of neurons. In aging and in AD, a decrease in the expression of the enzyme is usually observed [30].
3	Cofilin-1; P45592, (COF1_RAT)	Cofilin exhibits pH-sensitive depolymerizing activity in F-actin upon binding to it.	CB is an essential protein for maintaining calcium homeostasis and preventing neuronal death. Research has shown that removing CB from the amyloid precursor protein presenilin in transgenic mice worsens the pathogenesis of AD. This suggests its key role [31].
4	Ubiquitin carboxyl-terminal hydrolase isozyme L1 Q00981 (UCHL1_RAT)	UCHL1_RAT is a thiol protease. It hydrolyzes a peptide bond at the C-terminal glycine of ubiquitin.	There is evidence that UCH L1 binds and co-localizes with monoubiquitin and prolongs the half-life of ubiquitin. Uch-L1 reduction is part of a cycle that favors Aβ accumulation in vascular injury [32].
5	Calbindin P07171 (CALB1_RAT)	Calbindin (CB) buffers cytosolic calcium. It can stimulate membrane Ca^2+^-ATPase and 3’,5’-cyclic nucleotide phosphodiesterase.	CB is an essential protein for maintaining calcium homeostasis and preventing neuronal death. Research has shown that removing CB from the amyloid precursor protein presenilin in transgenic mice worsens the pathogenesis of AD. This suggests its key role [33].
6	Malate dehydrogenase, cytoplasmic O88989 (MDHC_RAT)	MDHC_RAT participates in the reduction in aromatic alpha-keto acids in the presence of NADH.	The enzyme is involved in oxidative phosphorylation by supplying NADH to the mitochondria. It has a key function in the Krebs cycle and the malate–aspartate shuttle. The functional significance of MDH elevation in AD is unknown [34].
7	Actin, cytoplasmic 1 P60711 (ACTB_RAT)	Actins are expressed in all eukaryotic cells and are involved in their motor processes.	A number of studies have shown that the actin cytoskeleton is essential for the function and plasticity of synapses. It is at the crossroads of the pathways between the amyloid cascade and synaptic dysfunction and therefore has a key role in AD pathogenesis [35].
8	Creatine kinase B-type P07335 (KCRB_RAT)	Creatine kinase (CK) is a transferase that catalyzes the reversible phosphate transfer reaction between ATP and various phosphogens.	CK plays a major role in the cellular energetics of the brain, so any disruption of this enzyme can worsen AD pathology. In AD patients, oxidation inactivates brain-type cytosolic creatine kinase (BB-CK) [36].
9	Glial fibrillary acidic protein P47819 (GFAP_RAT)	GFAP is a class III intermediate filament. It is a cell-specific marker that distinguishes astrocytes from other glial cells during the development of the central nervous system.	GFAP is a key protein and is a marker of astroglial damage. It is responsible for the structure of the cytoskeleton of glial cells, and for the regulation of the morphology and function of astrocytes, as well as for maintaining the blood–brain barrier. In AD, amyloid plaques are surrounded by reactive astrocytes with increased expression of GFAP filaments [37].
10	Tubulin alpha-1B chain Q6P9V9 (TBA1B_RAT)	Tubulin is the main building block of microtubules which are composed of α–β-tubulin heterodimers forming linear protofilaments that form a hollow polar cylinder.	Microtubules (MT) are essential components of the cytoskeleton of the cell, which has locomotory functions. A number of studies show that MT dysfunction may contribute to neurodegenerative processes and AD in particular. Disruption of the neuronal cytoskeleton is a feature of the neurodegenerative brain, and tubulin levels are decreased in the AD brain [38].
11	60 kDa heat shock protein, mitochondrial P63039 (CH60_RAT)	Hsp60 is a chaperone that is localized in the mitochondria and is involved in the correct folding of proteins.	Hsp60 is a protein that, together with Hsp10, is considered essential for mitochondrial protein folding. The role of Hsp60 in AD is still unclear [39].
12	V-type proton ATPase catalytic subunit A P50516 (VATA_MOUSE)	ATP synthase is the last enzyme of the mitochondrial electron transport chain, where oxidative phosphorylation takes place in order to synthesize ATP, which is a universal energy carrier in the cell.	In mammalian cells, ATP synthase, in addition to ATP synthesis, can also degrade (ATPase), which indicates the important function of this enzyme in the regulation of cellular metabolism and bioenergetics. The mechanisms of bioenergetic dysfunction, including the dysregulation of ATP synthase in AD, remain unclear [40].
13	Tropomyosin beta chain P58775 (TPM2_RAT)	Tropomyosin (Tm) binds to actin filaments in the cells. In non-muscle cells, it is involved in the stabilization of cytoskeletal actin filaments.	Tm has been shown to play an essential role in neurofibrillary pathology in AD. However, the exact role of Tm in AD pathology is still not well understood [41].
14	14-3-3 protein zeta/delta P63102 (1433Z_RAT)	14-3-3 is involved in the regulation of a broad spectrum of general and specialized signaling pathways. Binding usually results in a modulation of the activity of the protein to which 14-3-3 binds.	14-3-3 isoforms regulate a wide range of cellular processes, such as the cell cycle, transcription, intracellular trafficking, apoptosis, and autophagy [42]. In AD, they are thought to contribute to NFT formation through τ-hyperphosphorylation [43].
15	Kinesin-1 heavy chain Q2PQA9 (KINH_RAT)	Kinesin is a microtubule-dependent motor protein that participates in the normal distribution of mitochondria and lysosomes. It has a key role in the anterograde axonal transport of MAPK8IP3/JIP3, which is involved in axon elongation [44]	Studies have shown that the Kinesin-1 heavy chain is part of a key molecular motor protein involved in τ-homeostasis. It has been proposed that a reduction in Kinesin-1 heavy chain levels is sufficient to prevent and/or delay tau pathology in AD and other tauopathies [45].
16	Stathmin-4 P63043 (STMN4_RAT)	Stathmin (STM) is a ubiquitous cytosolic phosphoprotein primarily expressed in the nervous system and a member of a family of phosphoproteins that bind to tubulin and destabilize MTs.	STM-4 in the unphosphorylated or hypophosphorylated state binds to tubulin and prevents its polymerization, thereby preventing MTs assembly. After phosphorylation, STM-4 is released from tubulin and allows for the formation of MTs. The dysregulation of STM and MTs dynamics has been observed in aged animals and in patients with AD and depression [46].
17	Hemoglobin subunit alpha-1/2P01946 (HBA_RAT)	Hemoglobin participates in the transfer of oxygen from the lung to the various peripheral tissues.	According to research, hemoglobin (Hb) binds to Aβ and is found together with plaques and vascular amyloid deposits in the the brains of AD patients after death. Research by Chuang et al., 2012, suggests that the genesis of some plaques may be a consequence of prolonged amyloid accumulation at sites of vascular injury [47].

## Data Availability

Data are contained within the article.
